# Molecular Evidence of Breast Cancer Cell Proliferation Inhibition by a Combination of Selected Qatari Medicinal Plants Crude Extracts

**DOI:** 10.3390/nu15194276

**Published:** 2023-10-07

**Authors:** Nouralhuda Alateyah, Mohammed Alsafran, Kamal Usman, Allal Ouhtit

**Affiliations:** 1 Biological Sciences Program, Department of Biological & Environmental Sciences, College of Arts and Science, Qatar University, Doha P.O. Box 2713, Qatar; na1601400@student.qu.edu.qa (N.A.); m.alsafran@qu.edu.qa (M.A.); 2 Agricultural Research Station (ARS), Office of VP for Research & Graduate Studies, Qatar University, Doha P.O. Box 2713, Qatar; kusman@qu.edu.qa

**Keywords:** breast tumors, cell growth and cell survival, cell death, cell motility and invasion

## Abstract

Breast cancer (BC) is the most common malignancy, and conventional medicine has failed to establish efficient treatment modalities. Conventional medicine failed due to lack of knowledge of the mechanisms that underpin the onset and metastasis of tumors, as well as resistance to treatment regimen. However, Complementary and Alternative medicine (CAM) modalities are currently drawing the attention of both the public and health professionals. Our study examined the effect of a super-combination (SC) of crude extracts, which were isolated from three selected Qatari medicinal plants, on the proliferation, motility and death of BC cells. Our results revealed that SC attenuated cell growth and caused the cell death of MDA-MB-231 cancer cells when compared to human normal neonatal fibroblast cells. On the other hand, functional assays showed that SC reduced BC cell migration and invasion, respectively. SC-inhibited cell cycle and SC-regulated apoptosis was most likely mediated by p53/p21 pathway and p53-regulated Bax/BCL-2/Caspace-3 pathway. Our ongoing experiments aim to validate these in vitro findings in vivo using a BC-Xenograft mouse model. These findings support our hypothesis that SC inhibited BC cell proliferation and induced apoptosis. These findings lay the foundation for further experiments, aiming to validate SC as an effective chemoprevention and/or chemotherapeutic strategy that can ultimately pave the way towards translational research/clinical trials for the eradication of BC.

## 1. Introduction

Breast cancer (BC), a serious global health issue, is the most prominent type of tumors in women [1,2]. Malignant breast tumors can metastasize locally or to distant organs through the invasion of cells from the primary tumor to distant sites [3]. There has been major progress in technology that has led to breakthroughs in cancer research, paving the way towards designing specific-targeted therapies against cancer. However, conventional medicine (CM) has failed to establish the ultimate cure for cancer. This failure is due to the poor understanding of the specific molecular mechanisms associated with tumor development within various groups of patients, drug resistance, and the failure of clinical trials and the current therapies in curing cancer. Hence, the field of Complementary and Alternative medicine (CAM) is drawing more attention, especially in countries where CAM has been practiced for a long time [4,5]. CAM treatment modalities use crude extracts isolated from various medicinal plants. Each of these crude extracts encompass a variety of compounds or phytochemicals known for their anti-cancer properties [6,7].

Various natural compounds isolated from medicinal plants, commonly found in Qatar and the Gulf region, have been extensively explored for their anti-cancer properties in inhibiting cell proliferation and inducing apoptosis of different cancer cell lines. These plants include *Artemisia herba-alba* [8], Aloe vera [9], *Ferula asafetida* [10], Frankincense (*Boswellia sacra*) [11], Sidr (*Ziziphus spina-christi*) [12,13], Dates (*Phoenix dactylifera*) [14].

The induction of apoptosis (programmed cell death) by medicinal plant extracts involves complex mechanisms. These mechanisms can vary depending on the specific plant and its chemical composition. The common mechanisms that are often associated with the induction of apoptosis by plant extracts include the disruption of the mitochondrial membrane potential and favoring the pro-apoptotic proteins over the anti-apoptotic proteins to trigger apoptosis [15]. Plants extracts can also inhibit the cell proliferation of cancer cells by inducing cell cycle arrest and apoptosis [16]. On the other hand, phytochemical compounds isolated from plant extract can also inhibit the PI3K/Akt or the NF-κB (Phosphatidylinositol 3-kinase/Alpha serine/therionine-protein Kinase, Nuclear Factor-Kappa B) survival signaling pathways, which are commonly dysregulated in cancer cells to promote cell survival [17,18,19].

For the last two decades, our laboratory has focused on identifying an effective combination of phytochemicals/bioactives that can inhibit tumor cell proliferation and induce cancer cell death. The combination of bioactives can target, simultaneously, different signaling pathways that control cell proliferation and apoptosis [20]. Thus, we examined the effect of a super-combination (SC) of phyto-bioactives present in crude extracts, which were isolated from three selected Qatari medicinal plants (*Haplophyllum tuberculatum* (A), *Erodium glaucophyllum* (B), and *Heliotropium bacciferum* (C)) on the cell growth, cell survival, cell motility and invasion of the BC cell line MDA-MB-231. The plants used in the present study were selected based on our preliminary work, as well as their anti-cancer, anti-inflammatory properties, their content of antioxidants and well-known active compounds [21,22,23,24,25,26]. Moreover, the expression levels of proteins associated with cell cycle control and apoptosis were determined to identify the potential molecular mechanisms underlying SC-induced cell death.

## 2. Materials and Methods

### 2.1. Plants Collection

Fresh wild ABC plant species growing in Qatar’s desert sandy soil were collected during April and June 2016. These plants were identified by the second author (AlSafran M.) according to the standard sampling guidelines for local Qatari Medicinal plants [27]. The samples were collected in a polyethylene bag, immediately transported to the laboratory, and kept at −80 °C. Later, the samples were ground into a fine powder using a coffee grinder and sieved through a 24-mesh sieve for homogeneity. Powdered materials were then kept at −20 °C and protected from light until use.

### 2.2. Plant Crude Extraction

Crude extracts were isolated from the plants in methanol, as previously described with slight modifications [28]. Firstly, stored plant samples were dried, then 2 g of dried material was ground into a powder and transferred into a 50 mL polyethylene centrifuge tube. Powdered material was then mixed in 30 mL methanol for 24 h at ambient temperature in water bath, followed by ultrasonic frequency (50–60 Hz) bath (FS100B, Decon Laboratories Ltd., Hove, UK) for 60 min at 25 °C and. Before sonication, the tubes were vortexed for 10–15 s to enhance the yield of the extracts. The extract was centrifuged (Centurion Scientific C2) for 10 min at 2000 rpm, and the supernatant was transferred to an empty tube for storage at 4 °C. Plant residues were re-extracted with 30 mL of methanol, using the same procedure after 48 h period. A rotary evaporator (Laborota 4000, Heidolph, Germany) was used to evaporate combined supernatants to dryness under vacuum at 40 °C. Afterwards, the extracted product was then dissolved in 4 mL methanol, transferred to an amber glass vial, and kept at 4 °C in the refrigerator until use.

### 2.3. Determination of Total Phenolic Contents

The total phenolic contents were measured by following Folin–Ciocalteau (FC) method [29], using plant (50 µL) extracts mixed with 2.5 mL of 1:10 Folin–Ciocalteau reagent. Briefly, upon adding saturated Na_2_CO_3_ (75 g/L) solution and incubation at 30 °C for 1.5 h with intermittent shaking, the resulting blue-colored solution was measured at 765 nm using a spectrophotometer (Lambda 25, Perkin Elmer, Waltham, MA, USA). The measurements were carried out using a calibration curve of gallic acid mixed in methanol, and total phenolic content was expressed as Gallic Acid Equivalent (GAE) in mg/g of dry weight (DW) of the sample.

### 2.4. Determination of Total Flavonoid Contents

The aluminum chloride method was used to determine the total flavonoid content, with a few modifications from Chia-Chi et al. (2002) [30]. Each plant extract (0.5 mL) was mixed separately with 95% ethanol (1.5 mL), 10% aluminum chloride (0.1 mL), 1 M potassium acetate (0.1 mL), and distilled water (2.8 mL). The calibration curve was established by preparing Quercetin (QE) solution into 80% ethanol at a concentration of 25–100 µg/mL. The absorbance of the reaction mixture was measured at 415 nm after incubation for 30 min at room temperature, using a spectrophotometer (Lambda 25, Perkin Elmer), and total flavonoid content was expressed as mg QE/g DW.

### 2.5. Cell Lines and Cell Culture

The highly metastatic aggressive triple negative BC cell MDA-MB-231 was obtained from the American Type Culture Collection (ATCC, Manassas, Virginia). The primary dermal normal human neonatal fibroblast (HDFn), a cell line isolated from neonatal dermis were obtained from Weill Cornell Medicine-Qatar [20,31,32]. The cells were supplemented with DMEM media containing 10% fetal bovine serum (FBS), 1% penicillin–streptomycin, and 5% L-glutamine and then cultured and incubated at 37 °C in a humidified incubator adjusted to 5% CO_2_. All experiments were carried out until the cells reached 60 to 70% confluency.

### 2.6. Alamar-Blue Cell Proliferation Assay

Approximately 10,000 cells in each cell line were seeded in 96-wells culture plates in triplicates using DMEM medium to encompass all the ingredients, as described above. The cells were treated either with individual compounds, the SC combination, or with DMSO as a control. Based on preliminary optimization experiments, MDA-MB-231 and HDFn cells were treated with 0.798 mg/mL SC combination (the SC contained A = 0.4 mg/mL, B = 0.26 mg/mL, and C = 0.138 mg/mL). The SC killed more than 50% of the MDA-MB-231 cells compared to normal cells. This concentration of the SC was then selected for all the remaining experiments.

Following SC treatment for 24 and 48 h and PBS washing, the cells were incubated in 10% Alamar-Blue reagent (Invitrogen, Thermo Fisher Scientific, Waltham, MA, USA) for 4 h, as we have previously described [8]. Fluorescence was measured at 560 nm and 590 nm TECAN infinite M200 plate reader (Männedorf, Switzerland). The rate of cell proliferation/cell viability was determined based on the fluorescence of SC-treated cells compared to the control cells.

### 2.7. Morphological Study

Upon treatment with the sub-optimal dose of the SC, both MDA-MB-231 and human fibroblast cells were monitored alive under the microscope (OPTIKA Microscopes, Ponteranica, Italy) at 24 and 48 h post treatment [31,32].

### 2.8. Wound Healing Assay

To determine the effect of the SC treatment on BC cell migration, wound healing assay was applied to MDA-MB-231 cells, as we have previously reported [31]. Briefly, the cells were incubated in 6-well plates, and prior to the treatment, a straight scratch was made in each well using a sterile pipette tip. Following removal of floating cells and debris in a wash with PBS, the cells were treated with 0.798 mg/mL of SC and monitored for 48 h. During the treatment, the cells were observed and photographed at different time points (0, 24, and 48 h) to determine the wound closure of the scratch. Images were examined using the ImageJ application, and the data were analyzed and plotted in graphs.

### 2.9. Invasion Assay

The effect of the SC treatment on BC cell invasion was examined by Boyden cell invasion assay using Matrigel-coated chambers, as we have previously reported [32]. Briefly, treated cells were seeded onto the upper chambers of Matrigel plates, while Dulbeccos Modified Eagle Medium (DMEM) containing 10% FBS was added to the bottom chamber to serve as chemoattractant. Following cell incubation for a period of 48 h, cells that did not invade through the Matrigel were gently washed with phosphate buffered saline (PBS) and removed from the upper chambers using a sterile cotton swab. In contrast, cells that invaded to the lower chambers were fixed with methanol and formaldehyde for 10 min and then stained in 0.5% crystal violet. Once the crystal violet stain was removed with PBS, the cells were observed under the microscope, and photos were taken for analysis of cell invasion. The percentage of the inhibition of cell invasion was determined for the triplicates using Image J software version 1.53t (NIH, LOCI, University of Wisconsin, Madison, WI, USA).

### 2.10. Western Blot Analysis

The molecular mechanisms by which the SC inhibited cell growth and induced cell death were investigated by analyzing the expression levels of proteins associated with cell cycle (p53 and p21) as well as apoptosis (p53, BCL-2, Bax, Casp3 and Casp7), respectively, using Western blot analysis, as we have reported [4,8,18]. Pursuant to this goal, protein lysates were isolated from the 48 h SC-treated MDA-MB-231 cells (0.798 mg/mL), and 30 μg of total cell lysates were separated and transferred to PVDF membranes. Immunoblotting was carried out by probing the membranes overnight with the primary antibodies, including anti-rabbit p53 (Cell Signaling Technology, CST #2527S) anti-rabbit Bax (Cell Signaling Technology, CST #5023S), anti-mouse BcCL-2 (Cell Signaling Technology, CST #15071S), anti-rabbit Casp3 (Cell Signaling Technology, CST #9662), anti-mouse Casp7 (CST# AF823), and anti-rabbit Casp7 (CST# AF823). The anti-rabbit β-actin (Cell Signaling Technology, CST #4970S) served as a loading control. Following the incubation of the membranes with the secondary antibody for 2 h, immunoreactivity was visualized using ECL substrate (Pierce Biotechnology, Rockford, IL, USA). Images obtained with iBright developer (Thermo Fisher Scientific, Waltham, MA, USA), were analyzed using Image J software for the relative amounts of protein expression.

### 2.11. Statistical Analysis

Data obtained from the comparison between treated and untreated cells in the triplicated experiments were analyzed using Statistical Package of the Social Sciences (SPSS) and presented as mean ± Standard Error of the Mean (S.E.M). Means were compared using *t*-test, and differences were considered significant when *p* < 0.05.

## 3. Results

### 3.1. Total Phenolic Contents

**Figure 1:**
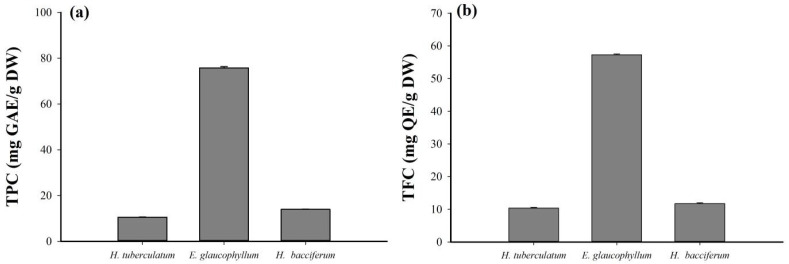
Total phenolic and flavonoid contents in the plants were used in the study. (**a**) Total phenolic contents (as mg gallic acid equivalents (GAE)/g of dry weight) and (**b**) total flavonoid content (as mg quercetin equivalents (QE)/g of dry weight) methanol extracts of selected plants.

Plant phenolic compounds have a variety of physiological activities [33], and many of these compounds are used in pharmaceuticals. Phenolic compounds exhibit health-improving properties, including reducing inflammation, free radicals, and cancer risk [34]. Figure 1a shows the total phenolic contents (TPC) measured using the FC method for the extracts with GAE as a standard sample. TPC values were calculated based on the calibration curve y = 0.0042x − 0.0005 R^2^ = 0.9993. It is calculated from the absorbance “x” and the concentration of the solution “y” (μg/mL) expressed as mg GAE/g DW. The TPC values were 10.55 mg GAE/g DW, 75.76 mg GAE/g DW, 13.97 mg GAE/g DW for *Haplophyllum tuberculatum*, *Erodium glaucophyllum* and *Heliotropium bacciferum*, respectively. Previous studies have reported the TPC in *E. glaucophyllum* [35], *H. bacciferum* [36], and *H. tuberculatum* [37].

### 3.2. Total Flavonoid Contents (TFC)

Flavonoids, which comprise the most abundant phenolic compound group in nature, are potent antioxidant, antimicrobial, antiulcer, anti-diabetic, hepato-protective, and anticarcinogenic agents [36]. A colorimetric assay was performed to evaluate the total flavonoid content (TFC) with QE as a standard sample. TFC values were calculated based on the calibration curve y = 0.001x + 0.0012 R^2^ = 0.9994. It is calculated from the absorbance x and the solution concentration y (μg/mL) expressed as mg QE/g DW (Figure 1b). The TFC values were 10.42 mg QE/g DW, 57.28 mg QE/g DW, 11.75 mg QE/g DW) for *H. tuberculatum*, *E. glaucophyllum* and *H. bacciferum*, respectively.

### 3.3. Establishment of the Optimal Dose of the Super-Combination

**Figure 2:**
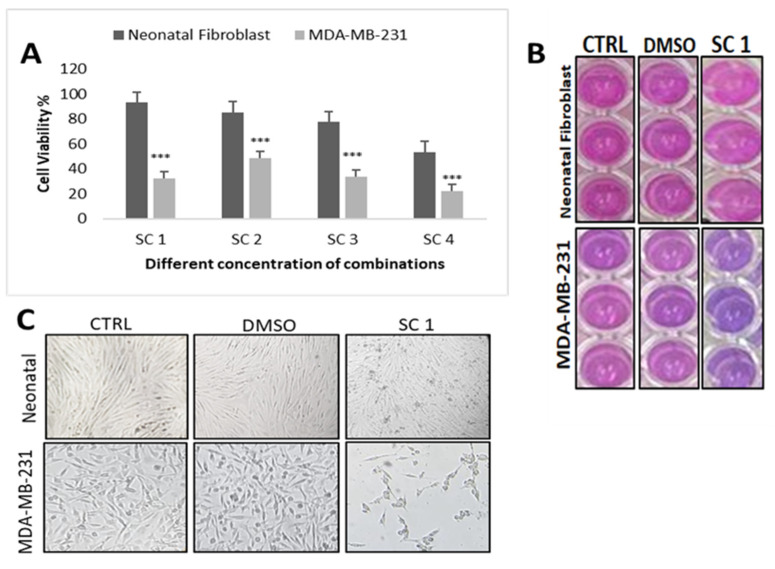
Effect of the optimal SC on breast cancer cell proliferation. (**A**,**C**) Effect of the SC on cell viability and morphology of MDA-231 cancer cells. (**B**) SC-treated cells on Alamar-blue colors (Pink: living cells; blue: dead cells). The results are statistically significant as *** *p* < 0.0001.

To determine the optimal dose of the super-combination (SC) that will be used in all experiments, (i) we determined the optimal dose for each individual crude extract isolated from each of the three selected Qatari medicinal plants (*H. tuberculatum* (A), *E. glaucophyllum* (B), and *H. bacciferum* (C)) by examining the effect of a range of ascending doses on the proliferation of MDA-MB-231 BC cell lines, and (ii) we examined the effect of different combinations (SC1 to SC4) on the growth of both MDA-MB-231 and the normal human dermal neonatal fibroblasts (HDF), which were used as control normal cells, while DMSO was used as vehicle control. Both cells were treated for 48 h with varying concentrations of SC (A + B + C), as shown in Table 1. Our results revealed that while the combinations tested all significantly inhibited MDA-MB-231 cell proliferation (Figure 2), SC1 showed the highest effect among all the SCs (Figure 2A).

### 3.4. Effect of the Super-Combination on the Cell Morphology

Next, we examined and compared the morphology of MDA-MB-231 cells and the human neonatal fibroblasts in the absence or presence of SC1 optimal dose. In the absence of SC1 treatment, MDA-MB-231 cells exhibited smooth epithelial with prominent nuclei. Compared to control cells, SC1-treated MDA-MB-231 lost cell–cell contact and detached from the tissue culture dish, suggesting SC1-induced cell death. More interestingly, however, the neonatal fibroblast control cells showed normal cell–cell contact and were healthy (Figure 2C).

### 3.5. Effect of the Super-Combination on the Cell Migration and Invasion

**Figure 3:**
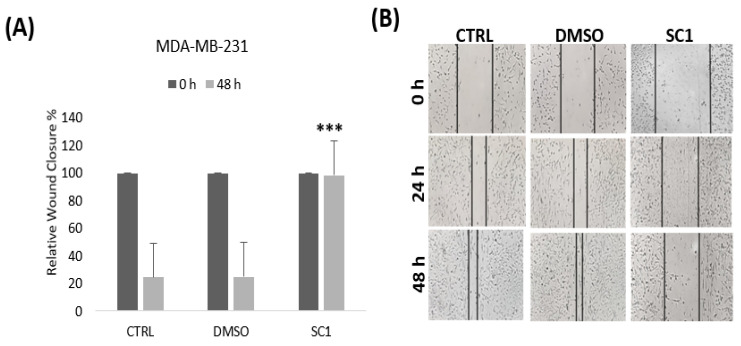
Effect of SC on MDA-MB-231 cell migration using scratch wound-healing assay. (**A**) SC1 inhibited MDA-MB-231 cell migration. (**B**) Semi-quantitative analysis of the relative wound closure (%) showing an increase in the wound closure by the SC, indicating an inhibition of MDA-231 cell migration. Representative images are mean values of the percentage of wound closure ± SEM (*n* = 3): *** *p* < 0.001.

**Figure 4:**
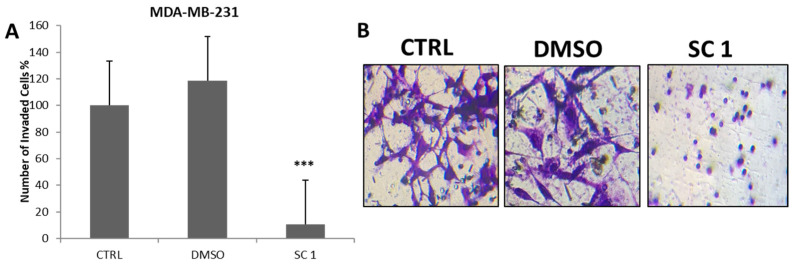
Effect of SC on MDA-MB-231 cell invasion using Boyden chamber assay. (**A**) Semi-quantitative analysis showing a decrease in invaded cells by the SC, indicating an inhibition of MDA-231 cell invasion. (**B**) Representative images of the invasion assay in MDA-MB-231 BC cells. SC 1 inhibited cell invasion of MDA-MB-231 by ~90% in comparison to control cells (*** *p* < 0.0001).

Next, we explored the effect of SC1 on BC cell migration and invasion using wound-healing and invasion Boyden chamber assays, respectively. Our results showed that SC1 significantly reduced BC cell migration (Figure 3) and invasion (Figure 4) by ∼73% (Figure 3) and 90% (Figure 4), respectively, when compared to the control (*p* < 0.05).

### 3.6. Molecular Mechanisms Mediating Super-Combination (SC)-Induced Apoptosis

**Figure 5:**
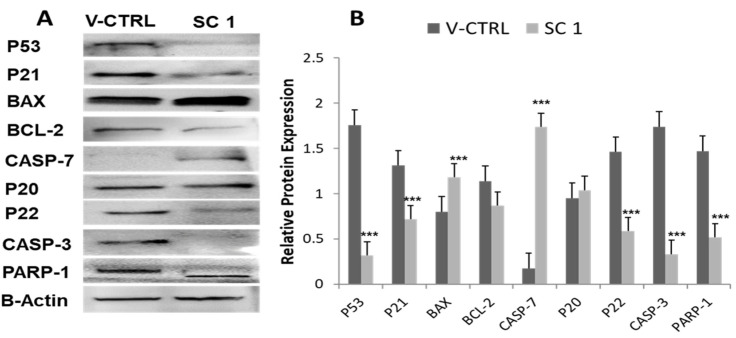
Expression levels of proteins associated with SC1-induced apoptosis. (**A**) Western blot analysis of SC1-inhibited mutant p53 expression, p21, BCL-2, and Casp3, while upregulating Bax and Casp7 compared to their vehicle control (V-CTRL). (**B**) Semi-quantitative analysis showing the relative expression level of each protein. β-actin served as control for the amount of proteins used in the assay. Density was analyzed using ImageJ. Mean Values ± SEM (*n* = 3) were compared using *t*-Test: *** *p* < 0.001.

Last but not least, we investigated the molecular mechanisms that underpin SC1-induced apoptosis. Therefore, after 48 h of SC treatment, protein lysates were collected from MDA-MB-231-treated cells as well as from their matched control DMSO untreated cells. The protein lysates were examined for the expression levels of the key genes associated with both cell cycle (p53 and p21) and apoptosis (p53, Bax, BCL-2, Casp3 and Casp7) using Western blot analysis (Figure 5). Interestingly, the expression of the mutant p53 was significantly attenuated by SC1 treatment in MDA-MB-231 as compared to the control cells (Figure 5A). Curiously, while MDA-MB-231 cells do express high levels of the stable mutant p53, the expression of p21 was remarkably inhibited, perhaps due to the inhibition of p53 (Figure 5A). However, SC1 significantly increased Bax and decreased BCL-2, indicating that, most likely, SC1 induced apoptosis via the intrinsic mitochondrial pathway by inducing Bax and inhibiting BCL-2 expression (Figure 5B). More interestingly, SC1 increased Casp7 but decreased Casp3, indicating that the apoptosis of MDA-MB-231 cells was executed via the induction of the effector Casp7, resulting in PARP-1 cleavage.

## 4. Discussion

The phenolic compounds in plants have a variety of physiological activities, and many of these compounds are used in pharmaceuticals. Phenolic compounds are believed to possess health-improving properties, such as reducing inflammation, reducing free radicals, and reducing cancer risk [20]. The TPC contents has already been reported in *E. glaucophyllum* [38], *H. bacciferum* [11], *H. tuberculatum* [36]. Several differences exist between the current study and earlier studies, including the methods of preparation, the extraction solvent, and the equivalent compounds. It is therefore not possible to compare TPC contents between the present study and earlier studies. Flavonoids comprise the most abundant phenolic compound group in nature; they have been shown to have diverse biological activities, including antioxidant, antimicrobial, antiulcer, anti-diabetic, hepato-protective, and anticarcinogenic properties [35].

Here, we explored the effects of the combination SC 1(SC1 = A + B + C) of crude extracts (Figure 2), isolated from three selected Qatari medicinal plants (*H. tuberculatum* (A), *E. glaucophyllum* (B), and *H. bacciferum* (C)), on cell proliferation, morphological changes, and the cell motility of the highly metastatic triple-negative BC cell line MDA-MB-231. The primary dermal normal human neonatal fibroblasts (HDFn) were used as control normal cells. Furthermore, we investigated the role of molecular mechanisms regulating the intrinsic pathway of apoptosis in SC-treated cells, as well as the expression of the key players mediating cell cycle and cell proliferation (Figure 3). Our findings revealed the SC-induced apoptosis and reduced cell migration and invasion of MDA-MB-231 when compared to normal control cells. More importantly, SC treatment resulted in apoptosis, which was most likely mediated by the mitochondrial Bax/BCL-2/Casp7 signaling pathway.

As indicated earlier, many studies have explored the medicinal potential of the plant crude extracts reported in this work or their active compounds, including their prospects as a source of cancer therapeutic agents. Although few of such studies investigated the anti-cancer potency of the individual crude extracts or their compounds, no study has investigated the combination of the crude extracts of the plant species used in our present investigation.

Several studies have isolated bioactive compounds and known active ingredients from plant species and characterized their anti-cancer properties. For instance, an analysis of crude extracts from *E. glaucophyllum* identified a number of known antioxidants bioactive, such as Geraniin [39,40,41], Gallocatechin [25], Quercetin and others [42]. An analysis of crude extracts from *H. bacciferum* showed the presence of a well-known carotenoid metabolite isololiolide [11,43]. More interestingly, the crude extracts from *H. tuberculatum* showed the presence of various alkaloids (e.g., haplotubinone, haplotubine, diphyllin, etc.) [44], polyphenols (e.g., lignans, arabelline, majidine, dictamine, and a qudsine derivative) [45,46], and flavonoids (e.g., resveratrol, kaempferol, myricetin, rutin, quercetin, etc.) [47].

It is evident from the literature described above that all the three medicinal plants (A, B and C) used in the present investigation are rich in various compounds (flavonoids, alkaloids, and polyphenols) that have already been characterized, individually, for their efficacy as anti-cancer agents. In fact, in our previous study, we combined in vitro cytotoxicity as well as microarray analysis, and identified, for the first time, a super-combination of six well-characterized bioactive compounds (resveratrol + indol-3-Carbinol + C-phycocyanin + isoflavone (genistein) + curcumin + quercetin), and showed that this super-cocktail induced apoptosis and inhibited the cell growth and motility of MDA-MB-231 BC cells [8]. More interestingly, our study revealed a myriad of major signaling pathways targeted by each of these individual compounds of the cocktail, in a combination, to exert synergistic anti-cancer activity [8]. Similarly, in the present study, it is obvious that mixing crude extracts from the three medicinal plants into an SC means a mix of various flavonoids, alkaloids, and polyphenols, as described above [11,43,44,45,46,47].

Several studies have already demonstrated the role of these compounds, individually, in inducing apoptosis and further inhibiting cancer cell migration and invasion (Figure 4). For instance, in addition to our previous studies on resveratrol and quercetin [8], geraniin, the main active ingredient isolated from *E. glaucophyllum*, was shown to inhibit human osteosarcoma cancer cell migration and invasion via the PI3K/Akt and ERK1/2 signaling pathways [39]. Furthermore, Geraniin decreased BCL-2 expression and increased Bax expression, leading to mitochondrial cytochrome c release and the subsequent activation of caspase-9 and caspase-3 cascades [39]. In vivo, geraniin resulted in tumor growth inhibition in A549 xenografts [39]. Isololiolide, which is abundant in *H. bacciferum*, induced significant apoptosis in the hepatocellular carcinoma HepG2 cells, when compared to non-malignant MRC-5 and HFF-1 human fibroblasts [43]. Isololiolide-induced apoptosis was associated with increased PARP cleavage and p53 expression and decreased procaspase-3 and BCL-2 levels [43]. In another study, lololide and isololiolide, isolated from *H. bacciferum,* were also found to inhibit the proliferation of colon cancer cells HCT116 and DLD1 [11]. Interestingly, Yang et al. reported that loliolide was a potent suppressor of the Epithelial-Mesenchymal Transition (EMT) process against colon and breast cancer cells [48]. Loliolide-suppressed EMT was associated with the inhibition of the chemokines CXCR4 and CXCR7, the suppression of expression of mesenchymal markers, and the induction of epithelial markers [48]. In addition, loliolide inhibited cancer cell invasion [48]. Based on the findings described above and the literature, the alkaloids and polyphenol compounds present in our crude extracts have been well characterized for their anti-cancer properties [49,50,51].

We also investigated the molecular mechanisms underlying SC-induced apoptosis and cell proliferation. Our results revealed that the SC attenuated cell growth and triggered apoptosis of the human TNBC cells most likely through the Bax/BCL-2 mitochondrial intrinsic pathway. As expected, and as shown in Figure 4, the SC led to the cleavage and activation of both Casp-3 and Casp-7, which in turn cleaved PARP-1, indicating that the SC induced the mitochondrial apoptosis of MDA-MB-231 BC cell lines. Interestingly, the SC inhibited the expression levels of the mutant p53 (Figure 4), while it induced Bax and inhibited BCL-2 (Figure 5). It must be noted that the stable mutant p53 is abundant in MDA-MB-231 cells and can suppress apoptosis via a dominant negative effect [52]. Also, the dysregulation of p53 function increases the Bax/BCL-2 ratio, rendering the cells vulnerable to apoptosis [53]. More importantly, regardless of the tumor suppression function of p53 [54], specific mutations confer a ‘gain-of-function’ or ‘dominant negative’ function to the new mutant p53 protein, which subsequently promote tumorigenesis and tumor progression [55,56,57]. Moreover, the abundance of mutant p53 protein in MDA-MB-231 [51] equip these cells with signals that promote cell survival by suppressing the pro-apoptotic effect that might originate from other p53 family members [52,53]. In summary, all our finding put together with the data described above from the literature support our hypothesis that the SC crude extract exhibited anti-cancer properties. In addition to its anti-migration/anti-invasive effects, it exhibited anti-proliferative effect, observed in MDA-MB-231, where it targeted and attenuated the expression of the mutant p53, subsequently resulting in apoptosis.

## 5. Conclusions

The findings support our hypothesis that SC inhibited cell proliferation, cell migration, cell invasion, and induced apoptosis. SC-promoted apoptosis appears to be mediated via the attenuation of the mutant p53 protein as well as upregulated Bax and downregulated BCL-2. This suggests that the super-combination triggered BC cell death via the p53/Bax/BCL-2/Casp7 pathway. Our ongoing experiments aim to validate these in vitro findings in vivo using a BC-Xenograft mouse model. These findings support our hypothesis that the SC of crude extracts exhibited promising anti-cancer properties and lays the foundation for further experiments to validate SC as an effective chemoprevention and/or chemotherapeutic strategy that can ultimately pave the way towards translational research/clinical trials for the eradication of BC.

## Figures and Tables

**Table 1:** The different concentrations of the super-combination (SC) examined on the proliferation of both MDA-MB-231 and the normal human dermal neonatal fibroblasts.

SC 1	A = 10%, B = 5%, C = 3%
SC 2	A = 10%, B = 10%, C = 3%
SC 3	A = 8%, B = 10%, C = 3%
SC 4	A = 5%, B = 10%, C = 4%

## Data Availability

All the data of the research is shown in the article. No other data is available.

## References

[1] Ferlay J., Colombet M., Soerjomataram I., Mathers C., Parkin D.M., Piñeros M., Znaor A., Bray F. (2019). Estimating the Global Cancer Incidence and Mortality in 2018: GLOBOCAN Sources and Methods. Int. J. Cancer.

[2] Narayan A.K., Al-Naemi H., Aly A., Kharita M.H., Khera R.D., Hajaj M., Rehani M.M. (2020). Breast Cancer Detection in Qatar: Evaluation of Mammography Image Quality Using A Standardized Assessment Tool. Eur. J. Breast Health.

[3] McSherry E.A., Donatello S., Hopkins A.M., McDonnell S. (2007). Molecular Basis of Invasion in Breast Cancer. Cell. Mol. Life Sci..

[4] Ouhtit A., Ismail M.F., Othman A., Fernando A., Abdraboh M.E., El-Kott A.F., Azab Y.A., Abdeen S.H., Gaur R.L., Gupta I. (2014). Chemoprevention of Rat Mammary Carcinogenesis by Spirulina. Am. J. Pathol..

[5] Longley D.B., Johnston P.G. (2005). Molecular Mechanisms of Drug Resistance. J. Pathol..

[6] Kawasaki B.T., Hurt E.M., Mistree T., Farrar W.L. (2008). Targeting Cancer Stem Cells with Phytochemicals. Mol. Interv..

[7] Moiseeva E.P., Manson M.M. (2009). Dietary Chemopreventive Phytochemicals: Too Little or Too Much?. Cancer Prev. Res..

[8] Khlifi D., Sghaier R.M., Amouri S., Laouini D., Hamdi M., Bouajila J. (2013). Composition and anti-oxidant, anti-cancer and anti-inflammatory activities of *Artemisia herba-alba*, *Ruta chalpensis* L. and *Peganum harmala* L.. Food Chem. Toxicol..

[9] Farshori N.N., Siddiqui M.A., Al-Oqail M.M., Al-Sheddi E.S., Al-Massarani S.M., Saquib Q., Ahmad J., Al-Khedhairy A.A. (2022). Aloe vera-induced apoptotic cell death through ROS generation, cell cycle arrest, and DNA damage in human breast cancer cells. Biologia.

[10] Alafnan A., Alamri A., Alanazi J., Hussain T. (2022). Farnesiferol C Exerts Antiproliferative Effects on Hepatocellular Carcinoma HepG2 Cells by Instigating ROS-Dependent Apoptotic Pathway. Pharmaceuticals.

[11] Azzazy H.M.E., Abdelnaser A., Al Mulla H., Sawy A.M., Shamma S.N., Elhusseiny M., Alwahibi S., Mahdy N.K., Fahmy S.A. (2022). Essential Oils Extracted from *Boswellia sacra* Oleo Gum Resin Loaded into PLGA-PCL Nanoparticles: Enhanced Cytotoxic and Apoptotic Effects against Breast Cancer Cells. ACS Omega.

[12] Qanash H., Bazaid A.S., Binsaleh N.K., Patel M., Althomali O.W., Sheeha B.B. (2023). In Vitro Antiproliferative Apoptosis Induction and Cell Cycle Arrest Potential of Saudi Sidr Honey against Colorectal Cancer. Nutrients..

[13] Hosmani J.V., Al Shahrani A., Hosmani J., AlShahrani I., Togoo R.A., Ain T.S., Syed S., Mannakandath M.L., Addas M.K.A. (2021). Cytotoxic and antitumor properties of Ziziphus spina-christi found in Al Bahah Region of Hejaz area: An in vitro study on oral cancer cell lines. Pharmacogn. Mag..

[14] Habib H.M., El-Fakharany E.M., El-Gendi H., El-Ziney M.G., El-Yazbi A.F., Ibrahim W.H. (2023). Palm Fruit (*Phoenix dactylifera* L.) Pollen Extract Inhibits Cancer Cell and Enzyme Activities and DNA and Protein Damage. Nutrients.

[15] Fouzat A., Hussein O.J., Gupta I., Al-Farsi H.F., Khalil A., Al Moustafa A.E. (2022). Elaeagnus, angustifolia Plant Extract Induces Apoptosis via P53 and Signal Transducer and Activator of Transcription 3 Signaling Pathways in Triple-Negative Breast Cancer Cells. Front. Nutr..

[16] Roudsari M.T., Bahrami A.R., Dehghani H., Iranshahi M., Matin M.M., Mahmoudi M. (2012). Bracken-fern extracts induce cell cycle arrest and apoptosis in certain cancer cell lines. Asian Pac. J. Cancer Prev..

[17] Nassan M.A., Soliman M.M., Ismail S.A., El-Shazly S. (2018). Effect of Taraxacum officinale extract on PI3K/Akt pathway in DMBA-induced breast cancer in albino rats. Biosci. Rep..

[18] Chen H., Zhu T., Huang X., Xu W., Di Z., Ma Y., Xue M., Bi S., Shen Y., Yu Y. (2023). Xanthatin suppresses proliferation and tumorigenicity of glioma cells through autophagy inhibition via activation of the PI3K-Akt-mTOR pathway. Pharmacol. Res. Perspect..

[19] Chen W., Li Z., Bai L., Lin Y. (2011). NF-kappaB in lung cancer, a carcinogenesis mediator and a prevention and therapy target. Front. Biosci..

[20] Ouhtit A., Gaur R.L., Abdraboh M., Ireland S.K., Rao P.N., Raj S.G., Al-Riyami H., Shanmuganathan S., Gupta I., Murthy S.N. (2014). Simultaneous Inhibition of Cell-Cycle, Proliferation, Survival, Metastatic Pathways and Induction of Apoptosis in Breast Cancer Cells by a Phytochemical Super-Cocktail: Genes That Underpin Its Mode of Action. J. Cancer.

[21] Kuete V., Wiench B., Alsaid M.S., Alyahya M.A., Fankam A.G., Shahat A.A., Efferth T. (2013). Cytotoxicity, Mode of Action and Antibacterial Activities of Selected Saudi Arabian Medicinal Plants. BMC Complement. Altern. Med..

[22] Eissa T.F., González-Burgos E., Carretero M.E., Gómez-Serranillos M.P. (2014). Biological Activity of HPLC-Characterized Ethanol Extract from the Aerial Parts of *Haplophyllum tuberculatum*. Pharm. Biol..

[23] Aïssaoui H., Mencherini T., Esposito T., De Tommasi N., Gazzerro P., Benayache S., Benayache F., Mekkiou R. (2019). *Heliotropium Bacciferum* Forssk. (Boraginaceae) Extracts: Chemical Constituents, Antioxidant Activity and Cytotoxic Effect in Human Cancer Cell Lines. Nat. Prod. Res..

[24] Abdallah B.M., Ali E.M. (2022). Therapeutic Effect of Green Synthesized Silver Nanoparticles Using *Erodium glaucophyllum* Extract against Oral Candidiasis: In Vitro and In Vivo Study. Molecules.

[25] Barba F.J., Alcántara C., Abdelkebir R., Bäuerl C., Pérez-Martínez G., Lorenzo J.M., Collado M.C., García-Pérez J.V. (2020). Ultrasonically-Assisted and Conventional Extraction from *Erodium glaucophyllum* Roots Using Ethanol:Water Mixtures: Phenolic Characterization, Antioxidant, and Anti-Inflammatory Activities. Molecules.

[26] Hamza G., Emna B.H., Yeddes W., Dhouafli Z., Moufida T.S., El Akrem H. (2018). Chemical Composition, Antimicrobial and Antioxidant Activities Data of Three Plants from Tunisia Region: *Erodium glaucophyllum*, *Erodium hirtum* and *Erodium guttatum*. Data Brief.

[27] Abdel Bary E.S.S. The Flora of Qatar: The Dicotyledons (Volume 1) and The Monocotyledons (Volume 2). https://www.chrome-extension://efaidnbmnnnibpcajpcglclefindmkaj/https://www.qu.edu.qa/static_file/qu/research/ESC/Books/The-Flora-of-Qatar-The-Dicotyledons.pdf.

[28] Stanković M.S., Petrović M., Godjevac D., Stevanović Z.D. (2015). Screening Inland Halophytes from the Central Balkan for Their Antioxidant Activity in Relation to Total Phenolic Compounds and Flavonoids: Are There Any Prospective Medicinal Plants?. J. Arid Environ..

[29] Singleton V.L., Rossi J.A. (1965). Colorimetry of Total Phenolics with Phosphomolybdic-Phosphotungstic Acid Reagents. Am. J. Enol. Vitic..

[30] Chang C.-C., Yang M.-H., Wen H.-M., Chern J.-C. (2002). Estimation of Total Flavonoid Content in Propolis by Two Complementary Colometric Methods. J. Food Drug Anal..

[31] Alateyah N., Ahmad S.M.S., Gupta I., Fouzat A., Thaher M.I., Das P., Al Moustafa A.E., Ouhtit A. (2022). Haematococcus Pluvialis Microalgae Extract Inhibits Proliferation, Invasion, and Induces Apoptosis in Breast Cancer Cells. Front. Nutr..

[32] Shariati S.R.P., Shokrgozar M.A., Vossoughi M., Eslamifar A. (2009). In vitro Co-Culture of Human Skin Keratinocytes and Fibroblasts on a Biocompatible and Biodegradable Scaffold. IBJ Iran. Biomed. J..

[33] Tsao R. (2010). Chemistry and biochemistry of dietary polyphenols. Nutrients.

[34] Ghasemzadeh A., Ghasemzadeh N. (2011). Flavonoids and Phenolic Acids: Role and Biochemical Activity in Plants and Human. J. Med. Plant Res..

[35] John B., Sulaiman C.T., George S., Reddy V.R.K. (2014). Total Phenolics and Flavonoids in Selected Medicinal Plants from Kerala. Int. J. Pharm. Pharm. Sci..

[36] Khasawneh M., Hamza A., Fawzi N. (2010). Antioxidant Activity and Phenolic content of Some Emirates Medicinal Plants. Adv. food Sci..

[37] Al-Brashdi A.S., Al-Ariymi H., Al Hashmi M., Khan S.A. (2016). Evaluation of Antioxidant Potential, Total Phenolic Content and Phytochemical Screening of Aerial Parts of a Folkloric Medicine, *Haplophyllum tuberculatum* (Forssk) A. Juss. J. Coast. Life Med..

[38] Gadhoumi H., Martinez-Rojas E., Tounsi M.S., Hayouni E.A. (2021). Phenolics Composition and Biological Activities Assessment of Leaves, Flowers and Roots Extracts from *Erodium glaucophyllum*, *Erodium hirtum* and *Erodium guttatum*. Biol. Bull..

[39] Wang Y., Wan D., Zhou R., Zhong W., Lu S., Chai Y. (2017). Geraniin Inhibits Migration and Invasion of Human Osteosarcoma Cancer Cells through Regulation of PI3K/Akt and ERK1/2 Signaling Pathways. Anticancer Drugs.

[40] Li J., Wang S., Yin J., Pan L. (2013). Geraniin Induces Apoptotic Cell Death in Human Lung Adenocarcinoma A549 Cells in Vitro and in Vivo. Can. J. Physiol. Pharmacol..

[41] Gohar A.A., Lahloub M.F., Niwa M. (2003). Antibacterial Polyphenol from *Erodium glaucophyllum*. Z. Naturforsch. C J. Biosci..

[42] Bakari S., Hajlaoui H., Daoud A., Mighri H., Ross-Garcia J.M., Gharsallah N., Kadri A. (2018). Phytochemicals, Antioxidant and Antimicrobial Potentials and LC-MS Analysis of Hydroalcoholic Extracts of Leaves and Flowers of *Erodium glaucophyllum* Collected from Tunisian Sahara. Food Sci. Technol..

[43] Vizetto-Duarte C., Custódio L., Gangadhar K.N., Lago J.H.G., Dias C., Matos A.M., Neng N., Nogueira J.M.F., Barreira L., Albericio F. (2016). Isololiolide, a Carotenoid Metabolite Isolated from the Brown Alga Cystoseira Tamariscifolia, Is Cytotoxic and Able to Induce Apoptosis in Hepatocarcinoma Cells through Caspase-3 Activation, Decreased BCL-2 Levels, Increased P53 Expression and PARP Cleavage. Phytomedicine.

[44] Al-Rehaily A.J., Al-Howiriny T.A., Ahmad M.S., Al-Yahya M.A., El-Feraly F.S., Hufford C.D., McPhail A.T. (2001). Alkaloids from *Haplophyllum tuberculatum*. Phytochemistry.

[45] Hamdi A., Viane J., Mahjoub M.A., Majouli K., Gad M.H.H., Kharbach M., Demeyer K., Marzouk Z., Heyden Y.V. (2018). Polyphenolic Contents, Antioxidant Activities and UPLC–ESI–MS Analysis of *Haplophyllum tuberculatum,* A. Juss Leaves Extracts. Int. J. Biol. Macromol..

[46] Mahmoud A.B., Danton O., Kaiser M., Han S., Moreno A., Algaffar S.A., Khalid S., Oh W.K., Hamburger M., Mäser P. (2020). Lignans, Amides, and Saponins from *Haplophyllum tuberculatum* and Their Antiprotozoal Activity. Molecules.

[47] Abdelkhalek A., Salem M.Z.M., Hafez E., Behiry S.I., Qari S.H. (2020). The Phytochemical, Antifungal, and First Report of the Antiviral Properties of Egyptian *Haplophyllum tuberculatum* Extract. Biology.

[48] Yang M.H., Ha I.J., Ahn J., Kim C.-K., Lee M., Ahn K.S. (2023). Potential Function of Loliolide as a Novel Blocker of Epithelial-Mesenchymal Transition in Colorectal and Breast Cancer Cells. Cell. Signal..

[49] Mondal A., Gandhi A., Fimognari C., Atanasov A.G., Bishayee A. (2019). Alkaloids for Cancer Prevention and Therapy: Current Progress and Future Perspectives. Eur. J. Pharmacol..

[50] Khan H., Alam W., Alsharif K.F., Aschner M., Pervez S., Saso L. (2022). Alkaloids and Colon Cancer: Molecular Mechanisms and Therapeutic Implications for Cell Cycle Arrest. Molecules.

[51] Cháirez-Ramírez M.H., de la Cruz-López K.G., García-Carrancá A. (2021). Polyphenols as Antitumor Agents Targeting Key Players in Cancer-Driving Signaling Pathways. Front. Pharmacol..

[52] Hui L., Zheng Y., Yan Y., Bargonetti J., Foster D.A. (2006). Mutant P53 in MDA-MB-231 Breast Cancer Cells Is Stabilized by Elevated Phospholipase D Activity and Contributes to Survival Signals Generated by Phospholipase D. Oncogene.

[53] Basu A., Haldar S. (1998). The Relationship between BCL-2, Bax and P53: Consequences for Cell Cycle Progression and Cell Death. Mol. Hum. Reprod..

[54] Levine A.J., Momand J., Finlay C.A. (1991). The P53 Tumour Suppressor Gene. Nature.

[55] Zambetti G.P., Levine A.J. (1993). A Comparison of the Biological Activities of Wild-type and Mutant P53. FASEB J..

[56] Blandino G., Levine A.J., Oren M. (1999). Mutant P53 Gain of Function: Differential Effects of Different P53 Mutants on Resistance of Cultured Cells to Chemotherapy. Oncogene.

[57] Cadwell C., Zambetti G.P. (2001). The Effects of Wild-Type P53 Tumor Suppressor Activity and Mutant P53 Gain-of-Function on Cell Growth. Gene.

